# Patch-Clamp Recording from Human Induced Pluripotent Stem Cell-Derived Cardiomyocytes: Improving Action Potential Characteristics through Dynamic Clamp

**DOI:** 10.3390/ijms18091873

**Published:** 2017-08-30

**Authors:** Arie O. Verkerk, Christiaan C. Veerman, Jan G. Zegers, Isabella Mengarelli, Connie R. Bezzina, Ronald Wilders

**Affiliations:** 1 Department of Medical Biology, Academic Medical Center, University of Amsterdam, 1105 AZ Amsterdam, The Netherlands; a.o.verkerk@amc.uva.nl (A.O.V.); j.g.zegers@amc.uva.nl (J.G.Z.); 2 Department of Experimental Cardiology, Academic Medical Center, University of Amsterdam, 1105 AZ Amsterdam, The Netherlands; c.c.veerman@amc.uva.nl (C.C.V.); i.mengarelli@amc.uva.nl (I.M.); c.r.bezzina@amc.uva.nl (C.R.B.)

**Keywords:** induced pluripotent stem cells, differentiation, retinoic acid, cardiomyocytes, electro-physiology, inward rectifier potassium current, perforated patch-clamp, computer simulations

## Abstract

Human induced pluripotent stem cell-derived cardiomyocytes (hiPSC-CMs) hold great promise for studying inherited cardiac arrhythmias and developing drug therapies to treat such arrhythmias. Unfortunately, until now, action potential (AP) measurements in hiPSC-CMs have been hampered by the virtual absence of the inward rectifier potassium current (*I*_K1_) in hiPSC-CMs, resulting in spontaneous activity and altered function of various depolarising and repolarising membrane currents. We assessed whether AP measurements in “ventricular-like” and “atrial-like” hiPSC-CMs could be improved through a simple, highly reproducible dynamic clamp approach to provide these cells with a substantial *I*_K1_ (computed in real time according to the actual membrane potential and injected through the patch-clamp pipette). APs were measured at 1 Hz using perforated patch-clamp methodology, both in control cells and in cells treated with all-trans retinoic acid (RA) during the differentiation process to increase the number of cells with atrial-like APs. RA-treated hiPSC-CMs displayed shorter APs than control hiPSC-CMs and this phenotype became more prominent upon addition of synthetic *I*_K1_ through dynamic clamp. Furthermore, the variability of several AP parameters decreased upon *I*_K1_ injection. Computer simulations with models of ventricular-like and atrial-like hiPSC-CMs demonstrated the importance of selecting an appropriate synthetic *I*_K1_. In conclusion, the dynamic clamp-based approach of *I*_K1_ injection has broad applicability for detailed AP measurements in hiPSC-CMs.

## 1. Introduction

The generation of human induced pluripotent stem cell-derived cardiomyocytes (hiPSC-CMs) holds great promise for studying inherited cardiac arrhythmias and for the development of drug therapies to treat such arrhythmias, as reviewed elsewhere, e.g., by Davis et al. [1] and Hoekstra et al. [2], and, more recently, by Kane and Terracciano [3] and Casini et al. [4]. To date, a variety of invasive and non-invasive methods are used for the electrophysiological analysis of hiPSC-CMs, including patch-clamp methodology, sharp electrode measurements, multi-electrode arrays (MEAs), and voltage-sensitive fluorescence, each with specific strengths and limitations [4]. However, reliable action potential (AP) measurements of hiPSC-CMs seem hampered by their intrinsic lack of inward rectifier potassium current (*I*_K1_) [5]. *I*_K1_ is the outward membrane current that modulates the final phase of the AP repolarisation and is essential for the generation of a stable resting membrane potential (RMP) close to the potassium equilibrium potential (*E*_K_) of approximately −85 mV. As a consequence of the virtual absence of *I*_K1_, hiPSC-CMs are frequently significantly depolarised and spontaneously active [6]. The altered AP configuration is associated with partial inactivation or slowed recovery from inactivation of the fast sodium current (*I*_Na_) [7,8], transient outward potassium current (*I*_to1_) [6,9] and L-type calcium current (*I*_Ca,L_) [10].

These days, many studies focus on maturation of hiPSC-CMs and thereby a natural increase in *I*_K1_ [6], e.g., by culturing hiPSC-CMs in 3D [11]. However, such procedures are not yet fully established and are certainly not common practice in electrophysiological studies on hiPSC-CMs [4]. In some studies on human embryonic stem cell-derived cardiomyocytes (hESC-CMs) or hiPSC-CMs *I*_K1_ was enhanced through adenoviral overexpression of the *I*_K1_ channel protein Kir2.1 (inward rectifier potassium channel subfamily 2 member 1) [12,13], whereas in other studies a stable RMP and more realistic AP configuration were obtained by “electronic expression” of *I*_K1_. The latter approach was followed by Bett et al. [14], Meijer van Putten et al. [5], and Rocchetti et al. [15], who all turned their patch-clamp system into a dynamic clamp system by injecting an *I*_K1_-like current through the patch-clamp pipette. In short, a synthetic *I*_K1_ is computed in real time based on the acquired membrane potential and injected into the hiPSC-CM through the patch-clamp pipette, thus hyperpolarising the cell and stabilizing its RMP and improving the resemblance with the AP configuration of atrial or ventricular cardiomyocytes. Another “electronic” solution to obtain an RMP near −80 mV, not requiring a dynamic clamp system, is to inject a hyperpolarising current of constant amplitude, as applied by, e.g., Jara-Avaca et al. [16].

Until now, most hiPSC-CMs studies have focused on ventricular arrhythmia syndromes, whereas atrial fibrillation (AF) is the most common type of arrhythmia, with a substantially increasing prevalence over the next decades [17,18]. Consequently, pharmacological agents targeting the atrial-specific K_v_1.5 and Kir3.1/3.4 potassium channels are being developed as new therapeutic strategies for AF [19,20]. Recently, we have shown that “atrial-like” hESC-CMs and hiPSC-CMs generated by modulating retinoic acid (RA) signalling provide a very promising pre-clinical model system to test pharmacological compounds for atrial selectivity [21] and study AF related arrhythmias due to K_v_1.5 dysfunction [22]. Due to the notorious difficulty in obtaining freshly isolated human atrial cardiomyocytes from patients [4], this human-based, physiologically relevant atrial hiPSC-CM model will likely be used more frequently in future for arrhythmia and drug discovery studies.

In the present study, we tested whether the application of an in silico *I*_K1_ also improved AP measurements in atrial-like hiPSC-CMs. We demonstrated that AP upstroke velocities, AP amplitudes and AP plateau amplitudes of both ventricular-like and atrial-like hiPSC-CMs increased upon the presence of an in silico *I*_K1_. Importantly, this synthetic *I*_K1_ reduced the variability in several AP parameters, which may allow detection of small changes in AP morphology due to genetic disorders and/or drugs. Finally, in presence of the synthetic *I*_K1_, the atrial-like AP phenotype is more pronounced, facilitating atrial-like vs. ventricular-like AP phenotype separation.

## 2. Results

### 2.1. Basic Action Potential Characterisation of Control and Retinoic Acid-Treated Human Induced Pluripotent Stem Cell-Derived Cardiomyocytes (hiPSC-CMs)

In the present study, we used single hiPSC-CMs that showed spontaneous beating upon visual inspection, clearly indicating a healthy and myocardial status. Typically for hiPSC-CMs, we observed a large variability in the frequency of the spontaneous beating. Both among control (CTRL) and RA-treated hiPSC-CMs, we observed cells with fast and highly regular beating, while other cells showed slow and less regular beating. The quickly beating cells displayed a clear diastolic depolarisation phase, while the more slowly beating cells exhibited an almost complete lack of diastolic depolarisation. We observed that the fast and highly regular beating hiPSC-CMs with a clear diastolic depolarisation phase all had a cycle length shorter than 700 ms, while the slow and irregular beating hiPSC-CMs all had a cycle length longer than 1000 ms. Therefore, we defined fast beating cells as having a cycle length shorter than 700 ms, while slow beating was defined as exhibiting a cycle length larger than 1000 ms. We analysed neither the ratio of fast versus slow beating cells in the complete cell population nor the mechanisms of pacemaking.

**Figure 1:**
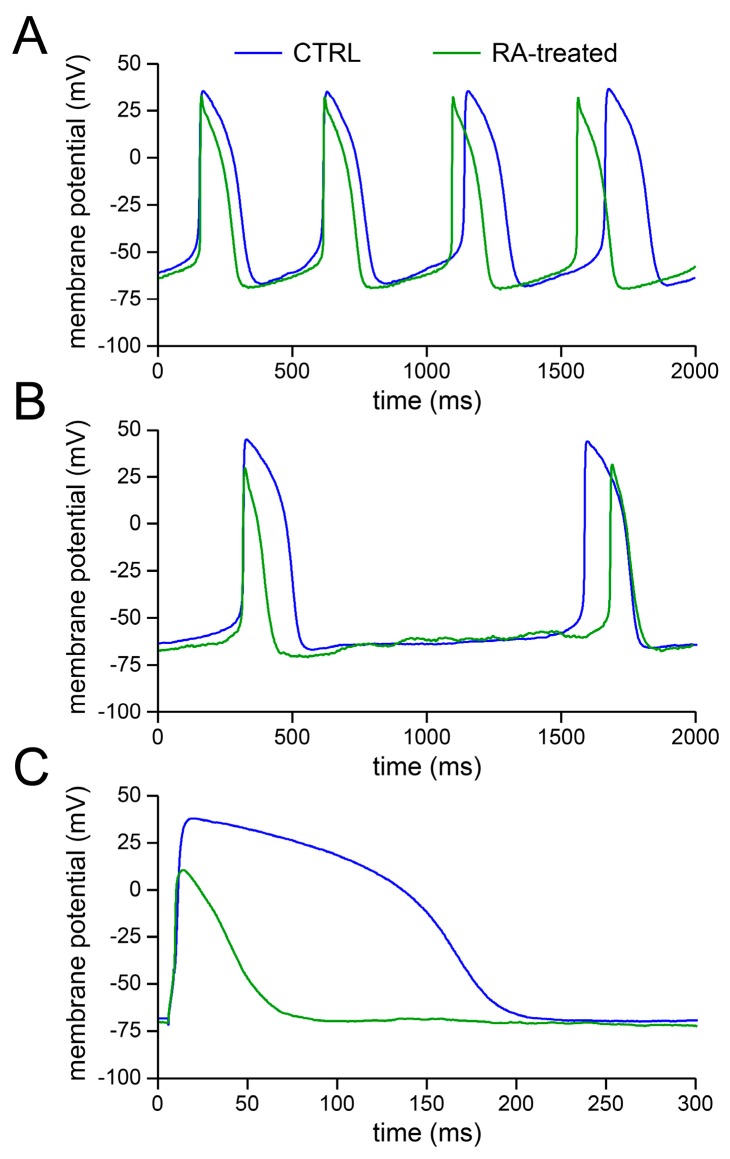
Superimposed action potentials of control (CTRL) and retinoic acid-treated (RA-treated) human induced pluripotent stem cell-derived cardiomyocytes (hiPSC-CMs). (**A**,**B**) Typical action potentials of (**A**) fast beating and (**B**) slowly beating CTRL and retinoic acid (RA)-treated hiPSC-CMs. (**C**) Typical action potentials during overdrive stimulation at 1 Hz. Note the expanded time scale.

Figure 1 shows typical AP recordings of the fast and slowly beating CTRL and RA-treated hiPSC-CMs after patching. The fast beating cells (Figure 1A) display a short cycle length and a clear diastolic depolarisation phase, while the slower beating cells (Figure 1B) have a longer cycle length and a virtually absent diastolic depolarisation phase. Of note, the long cycle length of the slowly beating cells allows overdrive stimulation at 1 Hz. Figure 1C shows typical action potentials elicited with such overdrive stimulation.

Table 1 summarises the average AP characteristics of CTRL and RA-treated cells of the fast and slowly beating groups. RA treatment resulted in a shorter action potential duration at 20% repolarisation (APD_20_) and lower AP plateau in both fast and slowly beating hiPSC-CMs. These changes in action potential parameters reflect the typical atrial-like AP phenotype (for review, see [23,24]) in the RA-treated group, consistent with previous findings [21,22].

### 2.2. Effects of I_K1_ Injection on Action Potential Morphology

**Figure 2:**
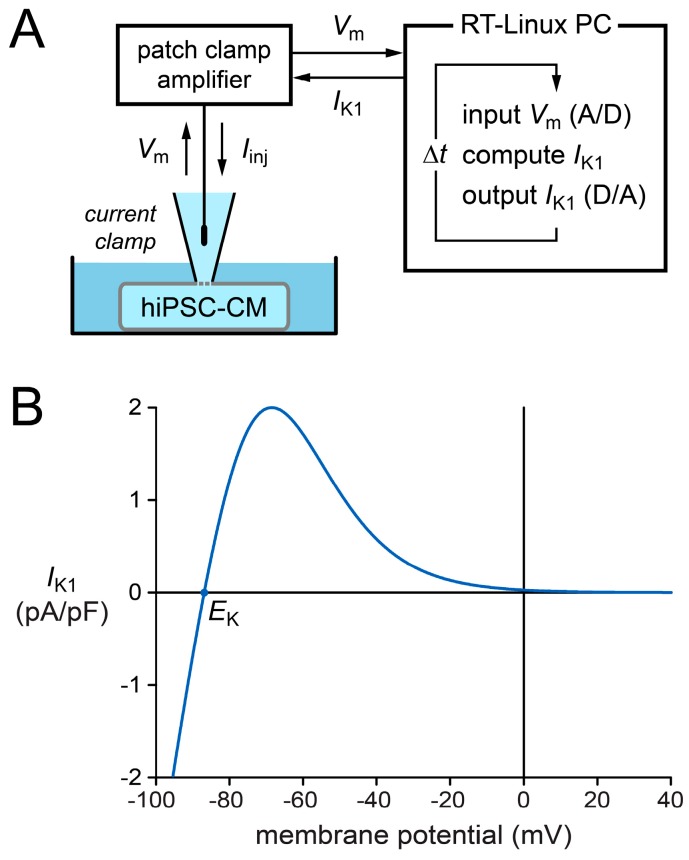
Experimental approach to supply a patched hiPSC-CM with a synthetic inward rectifier potassium current (*I*_K1_). (**A**) Diagram of the experimental setup. The Real-Time Linux (RT-Linux)-based PC computes the synthetic *I*_K1_ according to the membrane potential *V*_m_ that is read into the PC. This *I*_K1_ is sent to the patch-clamp amplifier, operating in current clamp mode, which adds any stimulus current and injects the net current (*I*_inj_) into the patched hiPSC-CM. This process is updated with a time step ∆*t*. (**B**) Current–voltage relationship of the synthetic *I*_K1_. *E*_K_ is the potassium equilibrium potential.

It was our aim to study the effects of *I*_K1_ injection on AP morphology in both CTRL and RA-treated hiPSC-CMs. To this end, we extended our patch-clamp setup with a separate Real-Time Linux (RT-Linux)-based PC that reads in the actual membrane potential *V*_m_ and computes the *V*_m_-dependent synthetic *I*_K1_. Within the time step ∆*t* of 40 µs, a command potential is generated that, combined with a command potential for any stimulus current, is sent to the patch-clamp amplifier to inject this current into the hiPSC-CM (Figure 2A).

For our synthetic *I*_K1_, we used the current–voltage relationship depicted in Figure 2B, which is based on data from Kir2.1 channels expressed in human embryonic kidney 293 (HEK-293) cells [25]. This approach was introduced by Meijer van Putten et al. [5] and was adopted in subsequent studies, e.g., those by Veerman et al. [26], Portero et al. [27], and Marczenke et al. [22]. As in these studies, we set the peak outward amplitude to 2 pA/pF.

We wanted to study the effects of *I*_K1_ injection without interference of differences in beating frequency. Therefore, we selected slowly beating cells that could be stimulated at an overdrive stimulus frequency of 1 Hz. As illustrated in Figure 1C above, which shows typical action potentials of a CTRL and an RA-treated cell upon overdrive stimulation at 1 Hz, the RA-treated cells showed a clear atrial-like phenotype. This is also apparent in the average values of the action potential parameters of both groups in the absence of *I*_K1_ injection, as listed in Table 2. The two groups differ in their action potential amplitude (APA), AP plateau amplitude (AP plateau), and action potential duration (APD) at 20%, 50%, and 90% repolarisation (APD_20_, APD_50_, and APD_90_, respectively).

**Figure 3:**
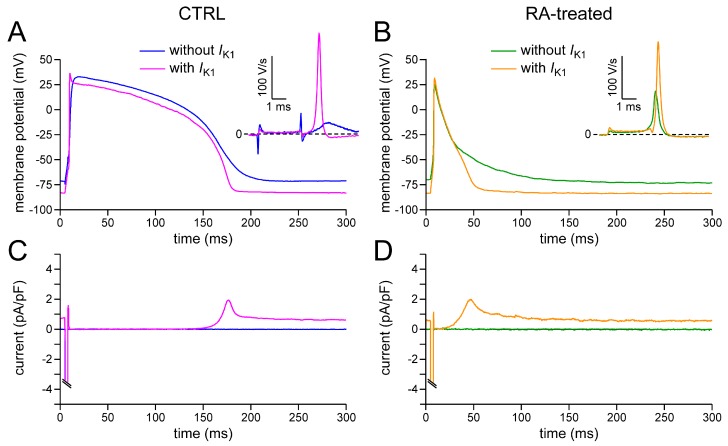
Effect of *I*_K1_ injection on the action potential of hiPSC-CMs. (**A**,**B**) Action potential of (**A**) a CTRL and (**B**) an RA-treated hiPSC-CM in the absence and presence of a synthetic *I*_K1_, which is computed in real time according to the current–voltage relationship of Figure 2B and supplied through the patch-camp pipette. Insets: time derivative of the AP upstroke. (**C**,**D**) Corresponding dynamic clamp current injected into the cell. The sharp cut-off peak of 3 ms duration starting at 5 ms represents the stimulus current, which is applied at 1 Hz.

We studied the effects of *I*_K1_ injection in a total of 13 CTRL and 18 RA-treated hiPSC-CMs. Figure 3A,B shows representative APs at 1 Hz stimulation from a CTRL and an RA-treated hiPSC-CM, respectively, both in the absence and presence of *I*_K1_ injection. The associated traces in Figure 3C,D show the injected current.

In the absence of *I*_K1_ injection, the action potential of the RA-treated hiPSC-CM repolarised earlier and faster than that of the CTRL cell, resulting in an action potential without a clear plateau phase, in line with the typical action potentials shown in Figure 1C and the average action potential parameters of Table 2. If the dynamic clamp system is switched on, it generates a synthetic *I*_K1_ with a peak outward current density of 2 pA/pF (Figure 3C,D) attained at a membrane potential of −69 mV (cf. Figure 3A,B), as expected from the *I*_K1_ current–voltage relationship of Figure 2B. Of note, the time course of the synthetic *I*_K1_ in Figure 3C is highly similar to the time course of *I*_K1_ observed by Jost et al. [28] in action potential clamp experiments on human ventricular tissue.

The *I*_K1_ injection has various effects on the action potential. First, it hyperpolarises the maximum diastolic potential (MDP) by 10–20 mV to a value near −83 mV (Figure 3A,B). At this potential, the synthetic *I*_K1_ is still substantial and amounts to 0.7–0.8 pA/pF (Figure 3C,D). With its steep current–voltage relationship (cf. Figure 2B) the synthetic *I*_K1_ creates a stable resting membrane potential close to the *E*_K_ of −87 mV. Second, it increases the maximum upstroke velocity (V_max_) substantially, as becomes evident from the insets in Figure 3A,B, which show the time derivative of the action potential traces during the AP upstroke. On average, V_max_ increases three-fold, from <100 V/s to >200 V/s (Table 2). This striking increase in V_max_, which reflects the underlying increase in *I*_Na_ [29], is the direct effect of the hyperpolarisation of the MDP to physiological values; the hyperpolarisation reduces the partial inactivation of *I*_Na_ at rest and also speeds up its recovery from inactivation. Third, the final AP repolarisation, which may be rather weak in the absence of synthetic *I*_K1_, is sped up by the additional outward current, both in the CTRL and the RA-treated hiPSC-CM (Figure 3A,B).

The effects of the *I*_K1_ injection on the action potential are reflected in the AP parameters that are listed in Table 2. The hyperpolarisation of the MDP and increase in V_max_ result in a prominent increase in the full amplitude of the AP and its plateau at 20 ms after initiation of the AP upstroke, both in CTRL and RA-treated hiPSC-CMs. Furthermore, the *I*_K1_ injection results in a significant increase in APD_20_, APD_50_, and APD_90_ in CTRL, but not in RA-treated hiPSC-CMs, thus accentuating the different AP morphologies of the CTRL and RA-treated hiPSC-CMs.

### 2.3. Variability in Action Potential Parameters

**Figure 4:**
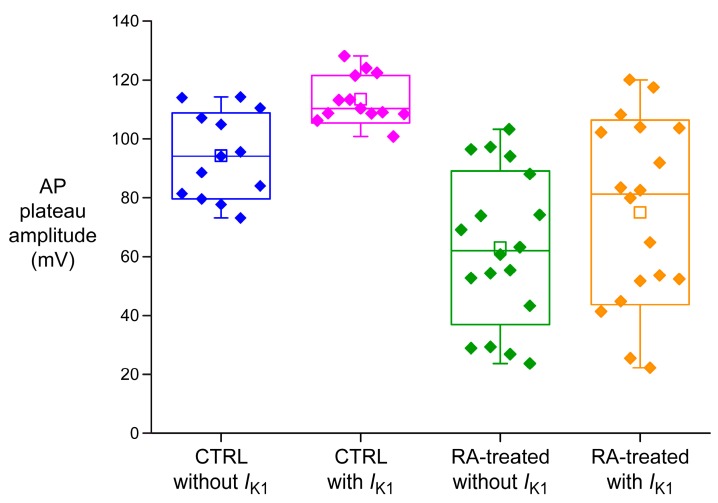
Standard box plot of the AP plateau amplitude of all CTRL and RA-treated hiPSC-CMs measured (*n* = 13 and *n* = 18, respectively), both with and without *I*_K1_ injection.

Although the average AP parameters of Table 2 underscore the atrial-like AP morphology of RA-treated hiPSC-CMs, there is a considerable heterogeneity in both the CTRL and RA-treated hiPSC-CM group, as illustrated in Figure 4, showing the AP plateau amplitude of all cells in a box plot. The heterogeneity is likely due to “natural” atrial-like hiPSC-CMs in the CTRL group, as observed frequently in hESC-CM and hiPSC-CM studies (for review, see [2]), and/or incomplete efficiency of the RA treatment.

**Figure 5:**
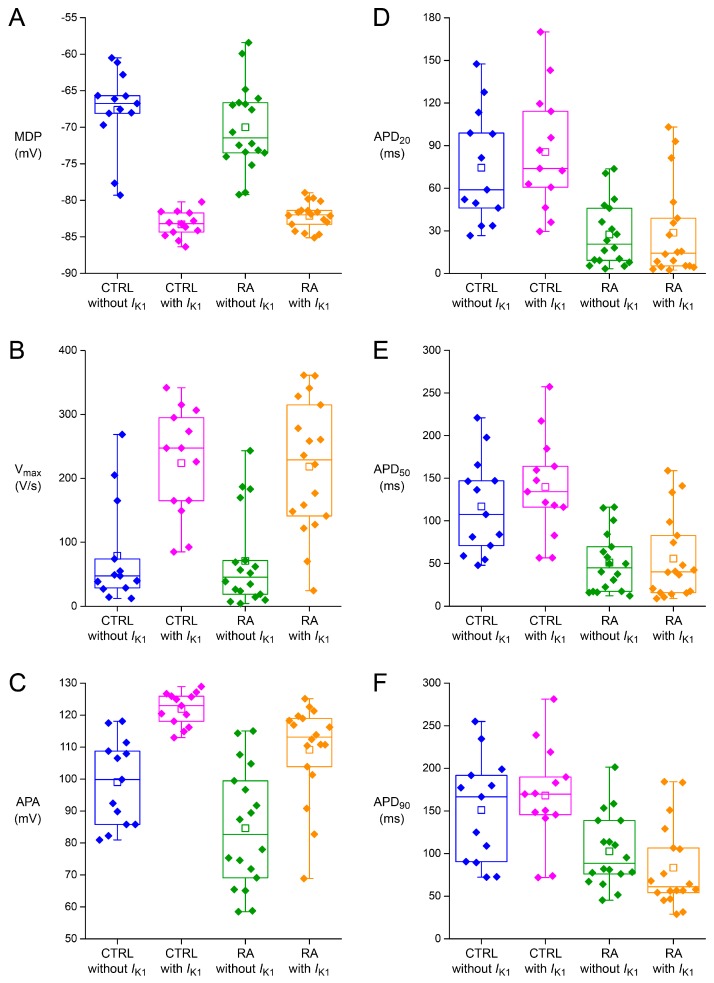
Standard box plots of action potential parameters of all CTRL and RA-treated hiPSC-CMs measured (*n* = 13 and *n* = 18, respectively), both with and without *I*_K1_ injection. (**A**) Maximum diastolic potential (MDP). (**B**) Maximum upstroke velocity (V_max_). (**C**) Action potential amplitude (APA). (**D**) Action potential duration at 20% repolarisation (APD_20_). (**E**) Action potential duration at 50% repolarisation (APD_50_). (**F**) Action potential duration at 90% repolarisation (APD_90_).

We anticipated that *I*_K1_ injection might reduce the variability in AP plateau amplitude by evoking a more natural AP upon “resetting” the MDP to a physiological value. This seems to hold true for the CTRL group (Figure 4), but not for the RA-treated group (Figure 4). A reduction in variability is important, because it would facilitate the detection of small changes in the AP configuration of hiPSC-CMs due to genetic disorders or drugs. We also assessed the variability in the other AP parameters. The results are shown as box plots in Figure 5.

Figure 5A not only shows the sensitivity of the MDP by 10–20 mV hyperpolarisation upon *I*_K1_ injection, but also demonstrates a marked reduction in MDP variability. This is encouraging, but not very surprising, because a synthetic *I*_K1_ of sufficient amplitude is expected to clamp the membrane potential at a value close to *E*_K_. In contrast, the variability in V_max_ seems to increase upon *I*_K1_ injection, in particular in case of the RA-treated hiPSC-CMs (Figure 5B). We have to keep in mind, however, that the apparent increase in variability may be largely due to the three-fold increase in V_max_ per se. The variability in APA decreases upon *I*_K1_ injection, but not so much for the RA-treated cells as for the CTRL cells (Figure 5C). It may be speculated that the former reflects a higher and more heterogeneous expression of *I*_to1_, which is a major determinant of APA, in the atrial-like RA-treated hiPSC-CMs.

The variability in action potential duration is shown in Figure 5D–F. Unfortunately, there is no clear reduction in variability, if any, upon *I*_K1_ injection. The box plots confirm the significant increase in APD_20_, APD_50_, and APD_90_ in case of the CTRL cells (Table 2), whereas the RA-treated cells do not exhibit such increase. Figure 5F even suggests that there is a trend towards a shorter APD_90_ of the RA-treated cells upon *I*_K1_ injection (*P* = 0.07), in line with the typical AP shown in Figure 3B.

**Figure 6:**
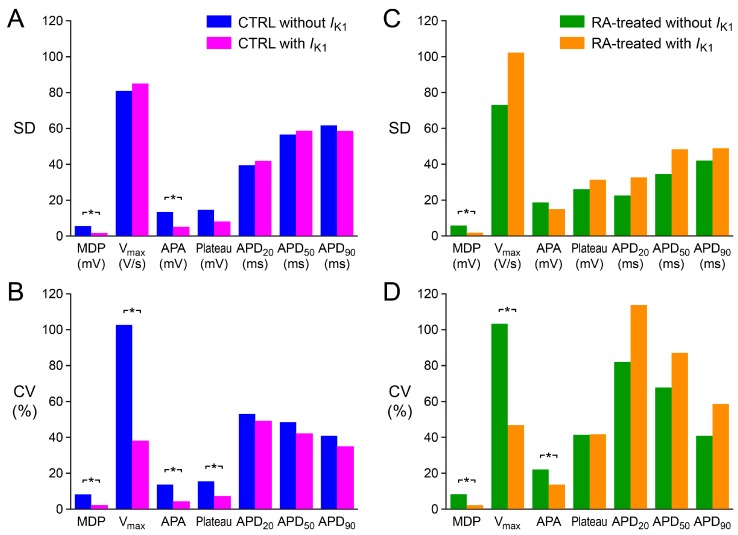
Variability in action potential parameters of the CTRL and RA-treated hiPSC-CMs, both with and without *I*_K1_ injection. (**A**) Standard deviation (SD) and (**B**) coefficient of variation (CV) of the AP parameters of the CTRL hiPSC-CMs. (**C**) SD and (**D**) CV of the AP parameters of the RA-treated hiPSC-CMs. MDP: maximum diastolic potential; V_max_: maximum upstroke velocity; APA: action potential amplitude; Plateau: AP plateau amplitude; APD_20_, APD_50_, and APD_90_: action potential duration at 20%, 50%, and 90% repolarisation, respectively. * *p* < 0.05.

The variability in AP parameters illustrated in Figure 4 and Figure 5 is quantified in Figure 6, which shows the standard deviation (SD) and coefficient of variation (CV), which is the ratio of the SD to the mean and is also known as relative standard deviation, for each of the AP parameters. The CTRL group shows a decrease in CV for all of the AP parameters (Figure 6B). The decrease is substantial for the MDP, V_max_, APA, and AP plateau amplitude, but only marginal and not statistically significant for APD_20_, APD_50_, and APD_90_; their CV is still ≈40%. The results are different for the RA-treated group. The CV is again reduced for the MDP, V_max_, and APA, but the CV of the AP plateau amplitude does not show any substantial change, whereas the CV is increased, although not statistically significant, for all action potential duration parameters (Figure 6D).

### 2.4. Assessing Dynamic Clamp Approach in Models of Ventricular-Like and Atrial-Like hiPSC-CMs

In the patch-clamp experiments described above, we employed an *I*_K1_ injection based on the Kir2.1-like current–voltage relationship of Figure 2B, which was introduced by Meijer van Putten et al. [5]. Bett et al. [14] and Rocchetti et al. [15] also employed an “electronic expression” of *I*_K1_ in their patch-clamp experiments on hiPSC-CMs, but they used different *I*_K1_ characteristics, as described in detail in Section 4.5. Others, like Jara-Avaca et al. [16], injected a hyperpolarising current of constant amplitude to obtain an RMP near −80 mV. We were interested to what extent the characteristics of the injected current would affect the outcome of the experiments. We tested this, but not in patch-clamp experiments on hiPSC-CMs, because we anticipated that the use of real hiPSC-CMs, with their considerable variability in AP morphology (Figure 4, Figure 5 and Figure 6), might lead to ambiguous results. Instead, we ran computer simulations with models of ventricular-like and atrial-like hiPSC-CMs [30,31], to which we added an outward current with the characteristics of the one used by Meijer van Putten et al. [5], Bett et al. [14], Rocchetti et al. [15], and Jara-Avaca et al. [16].

**Figure 7:**
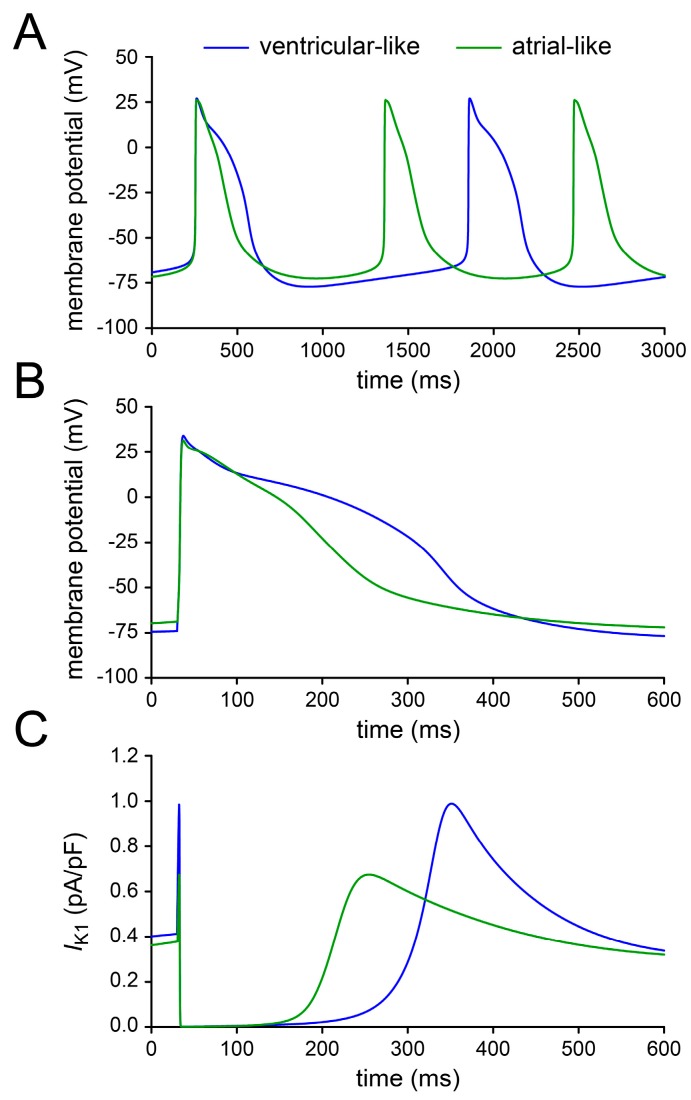
Electrical activity of the ventricular-like and atrial-like hiPSC-CM model cells of Paci et al. [30,31]. (**A**) Spontaneous action potentials. (**B**) Action potentials and (**C**) associated intrinsic inward rectifier potassium current (*I*_K1_) during 1 Hz overdrive stimulation on an expanded time scale.

Figure 7 shows the action potentials of the ventricular-like and atrial-like hiPSC-CM model cells (Figure 7A,B) and their intrinsic *I*_K1_ (Figure 7C), under control conditions, i.e., without any simulated current injection. Under control conditions, the ventricular-like and atrial-like hiPSC-CM model cells both show spontaneous action potentials with a cycle length >1000 ms (Figure 7A), like the slowly beating cells of Section 2.1, so that they can be stimulated at an overdrive frequency of 1 Hz. Figure 7B shows the APs, on an expanded time scale, that are obtained upon overdrive stimulation at 1 Hz. The action potential shape is similar to that of our CTRL and RA-treated hiPSC-CMs (cf. Figure 1C), albeit with a considerably longer AP duration. The time course of the intrinsic *I*_K1_ (Figure 7C) is highly similar to that of our injected *I*_K1_, with zero current during the AP plateau and ≈40% of its peak value at the RMP level (cf. Figure 3C,D). Indeed, the shape of the intrinsic *I*_K1_ current–voltage relationship [31] is quite similar to that of Meijer van Putten et al. [5]. Of note, the intrinsic *I*_K1_ of the model cells is rather large, in particular in the ventricular-like cell, if one takes into account that outward peak values of 0.6–2.2 pA/pF have been reported for human ventricular myocytes [28,32,33,34]. According to experimental data on the *I*_K1_ density at −100 mV, the *I*_K1_ density of hiPSC-CMs tends to be around 10 times smaller than that of human ventricular myocytes (see [5] and references cited therein). Yet, the relatively large intrinsic *I*_K1_ of the model cells is not sufficient to create an intrinsically quiescent cell with a stable RMP.

**Figure 8:**
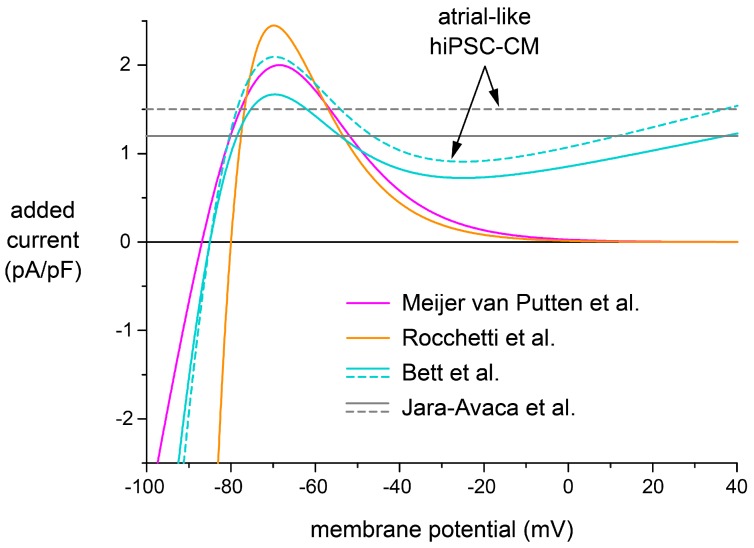
Current added to the ventricular-like and atrial-like hiPSC-CM model cells of Paci et al. [30,31] in order to obtain a resting membrane potential near −80 mV. *I*_K1_ current–voltage relationships of Meijer van Putten et al. [5], Bett et al. [14], and Rocchetti et al. [15] as well as the hyperpolarising current of Jara-Avaca et al. [16].

Figure 8 shows the *I*_K1_ current–voltage relationships of Meijer van Putten et al. [5], Bett et al. [14], and Rocchetti et al. [15] as well as the hyperpolarising current of Jara-Avaca et al. [16]. The current–voltage relationships employed by Meijer van Putten et al. [5] and Rocchetti et al. [15] appear to be highly similar. The main difference is the less negative *I*_K1_ reversal potential of −80 mV used by Rocchetti et al. [15]. Unlike these two Kir2.1-like *I*_K1_ current–voltage relationships, the *I*_K1_ of Bett et al. [14], which has a similar outward peak, does not approach zero at less negative membrane potential values, but attains a value around 1 pA/pF. This is because Bett et al. [14] intended to create a synthetic *I*_K1_ with a current–voltage relationship with characteristics of *I*_K1_ in both human atrial and ventricular myocytes as reported by Koumi et al. [35]. Furthermore, the *I*_K1_ density differs between the 99-pF ventricular-like and the 79-pF atrial-like model cells. This is because Bett et al. [14] used a fixed *I*_K1_ amplitude of 150 pA at −75 mV, independent of cell size. This results in a higher current density for the smaller atrial-like model cell (dashed “Bett et al.” trace in Figure 8).

All three *I*_K1_ formulations appeared sufficient to generate intrinsically quiescent ventricular-like and atrial-like hiPSC-CM model cells with a stable RMP near −80 mV (Table 3). In contrast with the dynamic clamp approach of Meijer van Putten et al. [5], Bett et al. [14], and Rocchetti et al. [15], Jara-Avaca et al. [16] injected a hyperpolarising current of constant amplitude into their hESC-CMs to obtain “physiological resting potentials (−80 mV)”. In the case of the ventricular-like model cell, an outward current of 1.2 pA/pF appeared sufficient to obtain a stable RMP near −80 mV, whereas the atrial cell required a 25% larger amplitude (Figure 8, solid and dashed horizontal lines). With these amplitudes, the “Jara-Avaca et al.” traces of Figure 8 are very similar to the “Bett et al.” traces at a membrane potential of −80 mV or above. However, the injection of a hyperpolarising current of constant amplitude may evoke an RMP near −80 mV in the absence of stimulation, but this does not ensure an appropriate diastolic potential during 1 Hz stimulation, as illustrated below.

### 2.5. Effects of Different I_K1_ Formulations on Action Potential Morphology

**Figure 9:**
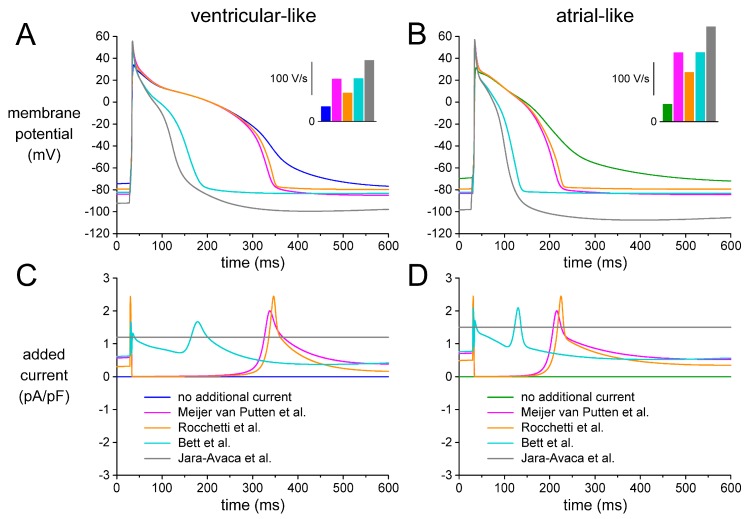
Electrical activity of simulated ventricular-like and atrial-like hiPSC-CMs. (**A**,**B**) Action potential during 1 Hz stimulation of (**A**) a ventricular-like and (**B**) an atrial-like model cell in the absence and presence of an additional *I*_K1_-like current, as applied by Meijer van Putten et al. [5], Bett et al. [14], or Rocchetti et al. [15], or a hyperpolarising current of constant amplitude, as applied by Jara-Avaca et al. [16]. Insets: maximum AP upstroke velocity. (**C**,**D**) Associated additional current.

We assessed the effects of each of the currents of Figure 8 on the electrical activity of the ventricular-like and atrial-like hiPSC-CM model cells upon stimulation at 1 Hz. Figure 9A,C shows the APs of the ventricular-like model cell and the associated current that was added to the cell model. The APs of the atrial-like model cell and associated current are shown in Figure 9B,D.

As in the experimental recordings of Figure 3, the main effects of adding the *I*_K1_ of Meijer van Putten et al. [5] or that of Rocchetti et al. [15] are a hyperpolarisation of the MDP, an increase in V_max_ (Figure 9A,B, insets) and a speeding up of the final AP repolarisation, both for the ventricular-like and the atrial-like hiPSC-CM model cell. The *I*_K1_ of Bett et al. [14] has similar effects, but it also shortens the APD dramatically (Figure 9A,B), due to the substantial *I*_K1_ at the AP plateau level (Figure 9C,D). The APD is even further shortened with the hyperpolarising current of Jara-Avaca et al. [16]. Furthermore, in the absence of the clamping effect of an *I*_K1_-like current, the MDP reaches values as negative as −100 and −107 mV (ventricular-like and atrial-like cell, respectively). The diastolic interval appears too short to let the model cells reach their resting potential near −80 mV, so that the AP takes off at −92 and −98 mV and a V_max_ of 186 and 289 V/s is achieved (ventricular-like and atrial-like cell, respectively).

Table 4 shows the AP parameters that we obtained with each of the synthetic *I*_K1_ formulations. We did not include data obtained with the injection of a hyperpolarising current of constant amplitude because this approach does not ensure an appropriate diastolic potential during the 1 Hz stimulation, as illustrated in Figure 9. The MDP values of Table 4, which were obtained during 1 Hz stimulation, are more negative than the resting membrane potentials listed in Table 3, which were obtained in the absence of stimulation. Table 4 shows considerable differences in APD_20_ between the Meijer van Putten et al. [5] and the Rocchetti et al. [15] *I*_K1_, which may not be appreciated from Figure 9. These differences reflect the sensitivity of this parameter to the AP amplitude, which is larger with the Meijer van Putten et al. [5] *I*_K1_ as a result of the more negative MDP and associated higher V_max_. This emphasises the importance of minimizing the variation in MDP through *I*_K1_ injection. Furthermore, the APD_20_, APD_50_, and APD_90_ values of Table 4 suggest that differences in APD between ventricular-like and atrial-like hiPSC-CMs may become obscured with the Bett et al. [14] synthetic *I*_K1_.

## 3. Discussion

### 3.1. Action Potential Morphology of CTRL and RA-Treated hiPSC-CMs and Underlying Ionic Currents

The action potentials that we recorded from ventricular-like hiPSC-CMs in the present study demonstrate a relatively short duration in comparison with those of freshly isolated adult human cardiomyocytes. However, comparison of action potential characteristics between different studies is often complicated due to differences in the employed experimental conditions (variable recording temperatures, perforated vs. whole-cell patch-clamp technique, spontaneously beating cells vs. triggered cells, absence or presence of Ca^2+^ buffers, etc.) [4]. Moreover, effects of regional differences in action potential morphology in human hearts and/or failing status cannot be ruled out [3]. Furthermore, hiPSC-CMs are thought to have an immature phenotype [6], and the membrane currents underlying their action potential may differ between hiPSC-CMs and freshly isolated adult human cardiomyocytes [2,4]. APD values vary widely between hiPSC-CMs studies [2,4]. Again, recording conditions may play a role [4], but additional factors such as the employed hiPSC-CMs line, differentiation and dissociation protocols, and the time interval between differentiation and AP recording may also contribute substantially to the apparent differences. Nevertheless, the APD values obtained in the present study are close to the values that we previously observed in our laboratory for control hESC-CMs and hiPSC-CMs [5,21,22,26,27,36,37,38,39,40]. Preliminary follow-up experiments demonstrate the virtual lack of a carbachol-activated current in our control hiPSC-CMs [41], which further demonstrates the ventricular nature of these cells, but opposes our speculation regarding the likely presence of “natural” atrial-like hiPSC-CMs in the CTRL group of our present study.

Our “atrial-like” RA-treated hiPSC-CMs exhibited a faster phase-1 repolarisation with a smaller APA and AP plateau amplitude than the control hiPSC-CMs, resulting in a relatively short AP. These differences in action potential morphology are in line with previous findings in RA-treated hESC-CMs [21] and hiPSC-CMs [22] and freshly isolated human myocytes [42,43]. Differences in ionic currents underlying the distinct AP morphology of atrial-like hESC-CMs and hiPSC-CMs have not been widely studied. Yet, the distinct AP morphology is at least related to differences in the expression of the ultrarapid delayed rectifier potassium current (*I*_Kur_) and the acetylcholine-activated potassium current (*I*_K,ACh_) [21,22], associated with the K_v_1.5 and Kir3.1/3.4 channel proteins, respectively.

Freshly isolated mammalian atrial and ventricular cardiomyocytes, including human myocytes, show differences in *I*_Na_, *I*_Ca,L_ and various repolarising membrane ionic currents. Sakakibara et al. [7,44] and Furukawa et al. [45] observed a highly similar *I*_Na_ in human atrial and ventricular cardiomyocytes. However, both Burashnikov et al. [46] and Calloe et al. [47] showed that atrial myocytes have a greater density of sodium channels than ventricular myocytes, but also a more negative half-inactivation voltage, which reduces their functional availability. Cohen and Lederer [48] found that the density of *I*_Ca,L_ was highly similar between human atrial and ventricular myocytes, but it turned out that *I*_Ca,L_ exhibits differences in its regulation by second messengers between atrial and ventricular myocytes, as reviewed by Hatem et al. [49]. In human myocytes, *I*_Kur_ was found in atrium but not in ventricle [42,43]. *I*_to1_ density was found to be similar, but inactivation of *I*_to1_ was more rapid and its steady-state inactivation occurred at more negative membrane potentials in atrial than ventricular cells [43]. In human ventricular myocytes, *I*_K1_ showed a much larger density and more pronounced rectification than in human atrial myocytes [33,35]. Similar observations were made in rabbit and canine atrium and ventricle [50,51]. Although Kir2.1 is by far the most abundant *I*_K1_ channel protein in both atrium and ventricle [32,52], these differences may be explained by differences in the localisation and regulation of the Kir2.x proteins between atrium and ventricle [52].

In recent years, it has become clear that sodium channel (Na_v_1.5) and inward rectifier potassium channel proteins (Kir2.1) reciprocally modulate their expression at the cardiomyocyte membrane through specific interactions within macromolecular complexes [53,54]. This reciprocal modulation of *I*_Na_ and *I*_K1_ channels is highly important in controlling cardiac excitability, as nicely demonstrated in a theoretical study by Varghese [55], but it is disturbed in hiPSC-CMs, given the presence of *I*_Na_ channels but virtual absence of *I*_K1_ channels in these cells. The presence of *I*_Na_ channels in hiPSC-CMs is often unnoticed because the majority of *I*_Na_ channels are inactivated at the considerably depolarised diastolic potential of hiPSC-CMs. However, as we demonstrated in the present study, injection of a synthetic *I*_K1_ can hyperpolarise the diastolic potential to physiological values near −80 mV and cause a striking increase in V_max_, thus unveiling the presence of functional *I*_Na_ channels. Further hyperpolarisation even increases the functional availability of *I*_Na_ channels. As emphasised by Goversen et al. [56], restoration of the reciprocal modulation of *I*_Na_ and *I*_K1_ channels might provide additional means to enhance Kir2.1 expression, and thus *I*_K1_, in hiPSC-CMs.

Assessment of the differences in membrane ionic currents underlying the differences in AP morphology of CTRL and RA-treated hiPSC-CMs was outside the scope of the present study. Further studies are required to elucidate the ionic mechanisms that underlie the AP morphology differences in detail. We previously demonstrated that *I*_Kur_ blockade by 50 µM 4-aminopyridine (4-AP) [21,22] or genetic manipulation [22] prolonged the APD_20_ of the atrial-like RA-treated hESC-CMs and hiPSC-CMs substantially. However, it did not become as large as that of the ventricular-like control cells. This finding contrasts slightly with data published in abstract form by Kaplan et al. [57]. They found that the APs of atrial-like hiPSC-CMs took on a ventricular-like shape upon blockade of *I*_Kur_ with 50 µM 4-AP. Moreover, they demonstrated, using dynamic clamp with a cloned K_v_1.5 current expressed in oocytes, that adding *I*_Kur_ to the ventricular myocytes changed their AP morphology from ventricular-like to atrial-like. In addition, they mentioned differences in *I*_Na_ magnitude and kinetics and most prominently in the transient outward currents [57].

### 3.2. Variability of Action Potential Parameters

Today, *I*_K1_ injection is routinely used in patch-clamp experiments on hiPSC-CMs in our laboratory [22,26,27]. This injection compensates for these myocytes’ intrinsic lack of *I*_K1_ and restores their physiological resting membrane potential, which in turn results in an improved AP morphology, as illustrated in Figure 3A, by “normalising” the electrophysiological role of major membrane ionic currents like *I*_Na_, *I*_to1_, and *I*_Ca,L_ [7,8,9,10]. We anticipated that the restoration of the AP morphology through *I*_K1_ injection might also reduce the variability in AP parameters. Such reduction in variability would facilitate the assessment of delicate effects of drugs and/or genetic disorders on the AP of hiPSC-CMs. Indeed, the variability in all seven AP parameters, quantified through their coefficient of variation, decreased in case of the ventricular-like CTRL hiPSC-CMs. The decrease was substantial for MDP, V_max_, APA and AP plateau, but only moderate and not statistically significant for APD_20_, APD_50_, and APD_90_ (Figure 6B). Unfortunately, the outcome was somewhat different in case of the atrial-like RA-treated hiPSC-CMs. The variability in MDP, V_max_, and APA again decreased substantially, but the variability in APD_20_, APD_50_, and APD_90_ increased rather than decreased, although not statistically significant (Figure 6D). One may speculate that this reflects a more heterogeneous nature of the RA-treated group, which in turn underlies the wide range of AP plateau amplitude values, both with and without *I*_K1_ injection (Figure 4). The wide range of AP plateau amplitude values without *I*_K1_ injection is in contrast with the observation by Devalla et al. [21] in hESC-CMs, also without *I*_K1_ injection, that the AP plateau amplitude of their RA-treated cells was typically <80 mV, whereas that of their control cells was >80 mV, and may point to a less efficient RA treatment in our hiPSC-CMs. It requires further research on our RA-treated hiPSC-CMs to find out if these cells are indeed less “atrial-like” than the RA-treated hESC-CMs of Devalla et al. [21].

Bett et al. [14] and Rocchetti et al. [15] also observed a decrease in variability of AP parameters upon *I*_K1_ injection in their hiPSC-CMs. Bett et al. [14] found a decrease in variability of the resting potential, APA and V_max_ (their Table 1), but their Figure 5E suggests that the variability in APD_90_ actually increased upon *I*_K1_ injection. The latter data were, however, obtained with only seven cells that exhibited an APD_90_ near 1 s and were stimulated at 0.25 Hz. In their “hiPSC-CMs with ventricular-like APs”, Rocchetti et al. [15] observed statistically significant changes in diastolic potential, APD_50_, APD_90_, and V_max_ upon *I*_K1_ injection, as shown in their Figure S11, but the error bars suggest that the decrease in variability of APD_50_ and APD_90_ is as moderate as in our ventricular-like CTRL hiPSC-CMs. A comprehensive dynamic clamp study on hiPSC-CMs by Marrus et al. [58], which has only been published in preprint form, also shows a moderate decrease in variability of APD_90_ upon *I*_K1_ injection.

*I*_K1_ injection may reduce the variability of action potential parameters, but the underlying fundamental problem of heterogeneity in hiPSC-CM populations remains. A further reduction of this heterogeneity, possibly in combination with synthetic *I*_K1_ injection, will allow the detection of small changes associated with genetic disorders and/or drugs. Also, the availability of less heterogeneous hiPSC-CM populations would facilitate the in vitro instead of in silico comparison of different *I*_K1_ formulations.

### 3.3. Selection of a Synthetic I_K1_

Our in silico experiments with ventricular-like and atrial-like hiPSC-CMs demonstrate that the AP morphology upon *I*_K1_ injection strongly depends on the characteristics of the selected synthetic *I*_K1_. Important parameters to take into account when selecting a synthetic *I*_K1_ are not only its current density, but also its reversal potential and its degree of rectification. In practical terms, the electrophysiologist in the lab should realise that turning the knob of the potentiometer to obtain a satisfactory resting potential may also affect AP duration (and other AP parameters).

The functional availability of *I*_Na_, and thereby the AP upstroke velocity, is largely determined by the diastolic potential, which is in turn determined by the *I*_K1_ reversal potential. With the *I*_K1_ of Rocchetti et al. [15] the diastolic potential is only a few mV less negative than with the *I*_K1_ of Meijer van Putten et al. [5] or Bett et al. [14], both with the ventricular-like and the atrial-like model cell (Figure 9A,B; Table 4). Yet, the difference in V_max_ is remarkable (Figure 9A,B, insets; Table 4).

In case of an *I*_K1_ with a strong Kir2.1-like rectification, like the *I*_K1_ of Meijer van Putten et al. [5] or Rocchetti et al. [15], and also that of Marrus et al. [58], the *I*_K1_ at the AP plateau level is negligible. With the more atrial-like *I*_K1_ of Bett et al. [14], which is used in the commercially available Cybercyte dynamic clamp system [59], the *I*_K1_ at the plateau level is no longer negligible and shortens the AP of both model cells dramatically (Figure 9A,B). This shortening effect will be less pronounced if the *I*_K1_ density is reduced for the atrial-like cell, in line with the experimental observation that the density of *I*_K1_ is lower in atrial myocytes [33,35]. However, further simulations demonstrate that the atrial-like model cell regains its spontaneous activity if the current density of the injected Bett et al. *I*_K1_ is reduced by 50% or more [60], despite the relatively large native *I*_K1_ of the atrial-like model cell.

The addition of a hyperpolarising current of constant amplitude to obtain a stable resting potential near −80 mV, as employed by Jara-Avaca et al. [16], also shortens the AP. The atrial-like cell requires a hyperpolarising current as large as 1.5 pA/pF, which is even larger than the 1.2 pA/pF required by the ventricular-like cell. Unlike *I*_K1_, the hyperpolarising current of constant amplitude lacks a reversal potential, thus letting the diastolic potential reach extremely negative values and creating an unsatisfactory AP morphology. The diastolic potential becomes less negative if the current amplitude is reduced, but lowering the amplitude beyond 1.45 pA/pF again results in loss of the stable resting potential [60].

The APD of our CTRL hiPSC-CMs, but not that of our RA-treated hiPSC-CMs, is prolonged upon *I*_K1_ injection (Table 2). This prolongation is in contrast with the study of Jost et al. [28], who observed an AP prolongation of 4.8 ± 1.5% in human right papillary muscle (and 17.9 ± 2.1% in dog) upon selective >70% inhibition of *I*_K1_ by 10 µM Ba^2+^. This contrasting finding may be related to the amount of change in MDP. In our experiments on hiPSC-CMs, the MDP was hyperpolarised by 10–20 mV upon *I*_K1_ injection, with secondary possibly AP prolonging changes in various membrane currents (including *I*_Na_, *I*_to1_, and *I*_Ca,L_), whereas the change in MDP upon (incomplete) I_K1_ block seems considerably less in the study of Jost et al. [28]. However, the AP prolongation of our CTRL hiPSC-CMs upon *I*_K1_ injection is also in contrast with the AP shortening upon *I*_K1_ injection that we observed in our computer simulations (Figure 9A).

### 3.4. Future Directions

Our study indicates that dynamic clamp can be easily used in manual patch-clamp experiments to optimise the AP recording from hiPSC-CMs. However, manual patch-clamp procedures can be complex and time-consuming, resulting in low throughput [4]. In contrast, automated patch-clamp can increase data throughput substantially by allowing multiple recordings of hiPSC-CMs in parallel [4]. It seems relatively easy to supplement automated patch-clamp with dynamic clamp, thus also optimizing AP recording from hiPSC-CMs in an automated patch-clamp setting. Yet, we have to keep in mind that automated patch-clamp systems have several technical limitations, as discussed elsewhere, e.g., by Yajuan et al. [61].

Recently, Quach and Christini [62] reported, in abstract form, on the development of a novel optical dynamic clamp method for use with hiPSC-CMs. They employed the hyperpolarising optogenetic tool ArchT [63] to supplement hiPSC-CMs with the lacking *I*_K1_. Although confirmation in vitro is still needed, in silico analysis predicted that addition of an *I*_K1_-like current via the precise activation of ArchT can be used to make hiPSC-CMs more electrophysiologically adult-like. This promising tool may be high-throughput because it allows for MEA measurements [64] as well as measurements with genetically engineered hiPSC-CMs expressing a voltage fluorescent indicator [65].

### 3.5. Limitations

In our patch-clamp experiments on hiPSC-CMs, we injected an in silico *I*_K1_ with a Kir2.1-like current–voltage relationship and a peak outward current density of 2 pA/pF, in line with data obtained on human ventricular myocytes. Such injection appeared sufficient to obtain a stable resting potential near −80 mV. This injected current was applied to both ventricular-like and atrial like cells, while, as mentioned above, the *I*_K1_ of human ventricular myocytes exhibits a much larger density and more pronounced rectification than that of human atrial myocytes [33,35]. Accordingly, as discussed above, the *I*_K1_ injected into atrial-like hiPSC-CMs should exhibit a weaker rectification and substantially smaller density. This is, however, not practically feasible. First, this requires a priori knowledge of the nature, i.e., ventricular-like vs. atrial like, of the patched cell. Second, as also observed in our computer simulations, the density of the injected *I*_K1_ can only be marginally reduced. A more substantial reduction of the *I*_K1_ density leads to depolarisation and spontaneous activity of the patched cells [5], probably by the presence of an immature and thus relatively large hyperpolarisation-activated cyclic nucleotide-gated funny current (*I*_f_) [2], thereby reducing the benefits of the injection of the lacking *I*_K1_. Thus, the injection of *I*_K1_ through dynamic clamp must be seen as a tool to optimise AP measurements rather than to simulate *I*_K1_ differences between atrial and ventricular myocytes in detail.

## 4. Materials and Methods

### 4.1. Differentiation to hiPSC-CMs

Human induced pluripotent stem cells (hiPSCs) were generated and characterised as described previously [5,66]. Next, adherent cultures of hiPSCs were adapted to feeder-free conditions on Matrigel-coated dishes in the presence of a chemically defined medium (E8 Essential Medium, Life Technologies, Bleiswijk, The Netherlands). Subsequently, differentiation of hiPSCs to CMs was performed in 20 days using Wnt signalling modulation by small molecule-based differentiation protocols in a serum-free, chemically defined medium [26,67]. Finally, we performed a metabolic selection-based enrichment for hiPSC-CMs by applying glucose-depleted culture medium containing 4 mM lactate for six days, thereby removing a large proportion of non-cardiomyocytes [68]. During the whole process, no serum was applied in the culture medium.

Previous experiments using this differentiation protocol and hiPSC-CM line demonstrated that the vast majority of the cells had a ventricular-like AP morphology [26]. To increase the amount of hiPSC-CMs with an atrial-like AP morphology, 1 µM all-trans retinoic acid (Sigma-Aldrich, Zwijndrecht, The Netherlands) was added from day 4 to 7 of differentiation, as described recently for hESC-CMs [21] and hiPSC-CMs [22].

### 4.2. Dissociation of hiPSC-CMs

For electrophysiological measurements, hiPSC-CMs were enzymatically dissociated from cultures into single cells. Therefore, patches of beating hiPSC-CMs were mechanically transferred from the cultures to a low-Ca^2+^ Tyrode solution, containing (in mM): 140 NaCl, 5.4 KCl, 0.01 CaCl_2_, 1.0 MgCl_2_, 5.5 glucose, 5.0 HEPES, and 14.1 creatine (pH 7.4; NaOH) and incubated for 10 min at room temperature. Subsequently, Liberase (0.04 mg/mL, Roche Chemicals, Sigma-Aldrich, Zwijndrecht, The Netherlands) and Elastase (0.01 mg/mL, Serva, Heidelberg, Germany) were added and cells were incubated for 10 min at 37 °C while shaking gently. Supernatant was then removed and a Kraft–Brühe solution (37 °C) was added to stop the enzymatic dissociation. This Kraft–Brühe solution contained (in mM): 85 KCl, 30 K_2_HPO_4_, 5.0 MgSO_4_, 5.5 glucose, 5.0 pyruvic acid, 5.0 creatine, 30 taurine, 5.0 Na-hydroxybutyric acid, 5.0 succinic acid, 2.0 Na_2_ATP and 1% BSA (pH 7.2; KOH). A short period of firm manual shaking followed by incubation at 37 °C for 10 min with shaking allowed for dissociation into single cells. The cells were then centrifuged and resuspended in a basic differentiation medium consisting of RPMI medium supplemented with B27 (Invitrogen, Bleiswijk, The Netherlands/Life Technologies), 20% FBS and penicillin/streptomycin (50 U/mL penicillin; 50 µg/mL streptomycin). The cell suspension was subsequently plated at a low density on Matrigel-coated coverslips. The medium was replaced with serum-free medium after 24 h and subsequently with antibiotic-free medium every 3–4 days. Electrophysiological experiments were performed 10–15 days after dissociation on spontaneously beating single cells.

### 4.3. Action Potential Measurements

APs were recorded at 36 ± 0.2 °C using an Axopatch 200B amplifier (Molecular Devices, Sunnyvale, CA, USA) and all potentials were corrected for the estimated liquid junction potential [69]. Data acquisition, voltage control, and analysis were accomplished using custom software. Pipettes (resistance 2–3 MΩ) were pulled from borosilicate glass capillaries using a vertical microelectrode puller. Cell membrane capacitance was estimated by dividing the time constant of the decay of the capacitive transient in response to 5 mV hyperpolarising voltage clamp steps from −40 mV by the series resistance. Signals were low-pass filtered with a cutoff frequency of 5 kHz and digitised at 40 kHz.

Action potentials were measured by the amphotericin-perforated patch-clamp methodology. Cells were superfused with a solution containing (in mM) 140 NaCl, 5.4 KCl, 1.8 CaCl_2_, 1.0 MgCl_2_, 5.5 glucose, and 5.0 HEPES (pH 7.4; NaOH). Pipettes were filled with a solution containing (in mM) 125 K-gluc, 20 KCl, 5.0 NaCl, 0.44 amphotericin-B, and 10 HEPES (pH 7.2; KOH). We recorded both spontaneous APs and APs that were elicited at 1 Hz by overdrive stimulation with 3 ms, 1.2 × threshold current pulses through the patch pipette. We analysed cycle length, maximum diastolic potential (MDP), maximum AP upstroke velocity (V_max_), AP amplitude (APA), AP plateau amplitude (measured 20 ms after initiation of the action potential upstroke), and AP duration at 20%, 50%, and 90% repolarisation (APD_20_, APD_50_, and APD_90_, respectively). Parameters from 10 consecutive APs were averaged.

### 4.4. Dynamic Clamp

To study the effects of an injected *I*_K1_ at a fixed stimulus frequency, we selected slowly beating cells that we stimulated at an overdrive stimulation of 1 Hz. We used a custom dynamic clamp setup to inject an in silico *I*_K1_ with a Kir2.1-like current–voltage relationship, as illustrated in Figure 2 and previously described in detail [5]. The *I*_K1_ current density (in pA/pF) is computed according to

(1) $$I_{K1}=0.25955\times \left(\frac{V_{m}-\;E_{K}}{1+\;e^{0.093633\times \left(V_{m}+72\right)}}\right),$$

in which *V*_m_ and *E*_K_ denote the membrane potential and potassium equilibrium potential (in mV), respectively, and *E*_K_ amounts to −86.9 mV. In the present study, we consistently used an *I*_K1_ peak outward amplitude of 2 pA/pF, as routinely employed in our laboratory [22,26,27], which resulted in quiescent hiPSC-CMs with an RMP of approximately −80 mV.

### 4.5. Computer Simulations

Single ventricular-like and atrial-like hiPSC-CMs were simulated using the ventricular-like and atrial-like versions of the hiPSC-CM model by Paci et al. [30,31]. We started from the CellML code of the 2013 model [30] as made available by Stefano Severi through the CellML Model Repository [70]. To edit and run the CellML code we used version 0.9.31.1409 of the Cellular Open Resource (COR) software developed by Alan Garny [71]. We modified the original “Paci2013” model [30] according to the slight modifications listed in the 2015 paper [31], thus arriving at the “Paci2015” model. We implemented an option to stimulate the cell at 1 Hz with an approximately 20% suprathreshold stimulus of 3 ms duration and we fixed the intracellular sodium concentration at its initial value to prevent any slow drift in the model variables. Furthermore, we incorporated an additional “injected” *I*_K1_, which could be computed according to the equations by Meijer van Putten et al. [5], Bett et al. [14], or Rocchetti et al. [15]. Also, we implemented an option to “inject” a hyperpolarising outward current of constant amplitude, as applied by Jara-Avaca et al. [16].

The Meijer van Putten et al. [5] *I*_K1_ was computed according to Equation (1) above. For the Bett et al. [14] *I*_K1_ (in pA), we used

(2) $$I_{K1}=96.81\times \left(0.5\times \left(\frac{V_{m}+85}{1+e^{0.0896\times \left(V_{m}+85\right)}}\right)+0.01\times \left(V_{m}+85\right)\right),$$

in which *V*_m_ denotes the membrane potential (in mV). We added the scaling factor of 96.81 to the original equation of Bett et al. [14] to arrive at their fixed outward current of 150 pA at −75 mV, which they obtained by setting a potentiometer in their experimental setup.

Rocchetti et al. [15] based their *I*_K1_ on the ORd (O’Hara–Rudy dynamic) model of an adult human ventricular myocyte [72]. Their equation for *I*_K1_ (in pA/pF) is:

(3) $$I_{K1}=G_{K1}\times \sqrt{{\left[K\right]}_{o}}\times \left(\frac{V_{m}-E_{K1}}{1+e^{\left(\frac{V_{m}+105.8-2.6\times {\left[K\right]}_{o}}{9.493}\right)}}\right),$$

in which *G*_K1_ is the *I*_K1_ conductance (in nS/pF), [K]*_o_* is the extracellular potassium concentration (in mM), and *V*_m_ and *E*_K1_ denote the membrane potential and *I*_K1_ reversal potential (in mV), respectively. Like Rocchetti et al. [15], we set *G_K_*_1_ to 1.9 nS/pF, *E_K_*_1_ to −80 mV, and [K]_o_ to 4 mM. We did not incorporate the ORd model-based time dependent inactivation of *I*_K1_ of Rocchetti et al. [15], thus allowing a direct comparison of the three different formulations. In the physiological voltage range, the effects of the time-dependent inactivation are negligible, given the small time constant of inactivation (typically < 10 ms) and the steady-state inactivation near 1 (>0.99) [15].

### 4.6. Statistics

Data are presented as mean ± SEM. Statistical analysis was carried out with SigmaStat 3.5 software (Systat Software, Inc., San Jose, CA, USA). Two groups were compared with a paired or unpaired t-test or, in case of a failing normality and/or equal variance test, a Wilcoxon Signed Rank Test or a Mann–Whitney Rank Sum Test, respectively. Normality and equal variance assumptions were tested with the Kolmogorov–Smirnov and Levene median test, respectively. Comparison of two (relative) standard deviations was performed by means of an F-test. *p* < 0.05 was considered statistically significant.

## 5. Conclusions

Patch-clamp recording of APs from hiPSC-CMs, both “ventricular-like” and “atrial-like”, can be optimised through dynamic clamp to provide these cells with a substantial *I*_K1_ and thus with a physiological resting membrane potential. This does not only restore the AP morphology, but also decreases the variability of AP parameters, thereby facilitating the detection of small changes in AP morphology due to genetic disorders and/or drugs.

## Figures and Tables

**Table 1:** Action potential parameters of spontaneously beating control and retinoic acid (RA)-treated human induced pluripotent stem cell-derived cardiomyocytes (hiPSC-CMs).

Action Potential Parameter	Fast Beating Cells		Slowly Beating Cells	
	CTRL (*n* = 9)	RA (*n* = 12)	CTRL (*n* = 9)	RA (*n* = 9)
MDP (mV)	−68.2 ± 1.8	−68.5 ± 0.6	−70.4 ± 2.8	−70.8 ± 1.3
Vmax (V/s)	85.3 ± 29.3	40.8 ± 9.9	84.1 ± 30.2	29.7 ± 5.9
APA (mV)	105.1 ± 3.8	94.7 ± 4.6	108.4 ± 5.8	82.2 ± 5.7 *
AP plateau (mV)	99.5 ± 4.2	86.1 ± 4.8 *	103.1 ± 4.0	72.1 ± 8.5 *
APD20 (ms)	78.1 ± 15.5	45.3 ± 5.7 *	91.5 ± 11.0	42.1 ± 8.7 *
APD50 (ms)	124.7 ± 21.9	81.5 ± 9.7	144.3 ± 17.9	79.2 ± 13.7 *
APD90 (ms)	161.7 ± 23.9	122.8 ± 13.9	181.7 ± 19.6	152.4 ± 34.2
Cycle length (ms)	565 ± 57	488 ± 50	1392 ± 163	2138 ± 502

Data are mean ± SEM. CTRL: control cells; RA: retinoic acid-treated cells; *n*: number of cells; MDP: maximum diastolic potential; V_max_: maximum upstroke velocity; APA: action potential amplitude; AP plateau: AP plateau amplitude; APD_20_, APD_50_, and APD_90_: action potential duration at 20%, 50%, and 90% repolarisation, respectively. * *p* < 0.05 RA vs. CTRL.

**Table 2:** Action potential parameters of control and RA-treated hiPSC-CMs, stimulated at an overdrive frequency of 1 Hz, in the absence or presence of *I*_K1_ injection.

Action Potential Parameter	CTRL (*n* = 13)		RA (*n* = 18)	
	without *I*_K1_	with *I*_K1_	without *I*_K1_	with *I*_K1_
MDP (mV)	−67.7 ± 1.6	−83.3 ± 0.5 ^†^	−70.0 ± 1.4	−82.1 ± 0.4 ^†^
V_max_ (V/s)	78.8 ± 23.4	233.8 ± 24.5 ^†^	70.7 ± 17.7	218.3 ± 24.8 ^†^
APA (mV)	99.0 ± 3.9	121.9 ± 1.5 ^†^	84.6 ± 4.5 *	109.2 ± 3.6 * ^†^
AP plateau (mV)	94.2 ± 4.2	113.5 ± 2.3 ^†^	63.1 ± 6.3 *	75.0 ± 7.6 * ^†^
APD_20_ (ms)	74.4 ± 11.4	85.5 ± 12.1 ^†^	27.4 ± 5.5 *	28.6 ± 7.9 *
APD_50_ (ms)	116.8 ± 16.4	139.6 ± 16.9 ^†^	50.9 ± 8.4 *	55.5 ± 11.7 *
APD_90_ (ms)	151.0 ± 17.8	168.0 ± 16.9 ^†^	102.6 ± 10.2 *	83.4 ± 11.9 *

Data are mean ± SEM. CTRL: control cells; RA: retinoic acid-treated cells; *n*: number of cells; MDP: maximum diastolic potential; V_max_: maximum upstroke velocity; APA: action potential amplitude; AP plateau: AP plateau amplitude; APD_20_, APD_50_, and APD_90_: action potential duration at 20%, 50%, and 90% repolarisation, respectively. * *p* < 0.05 RA vs. CTRL. ^†^ *p* < 0.05 with vs. without *I*_K1_ injection.

**Table 3:** Resting membrane potential evoked in hiPSC-CM model cell.

Experimental Approach	Resting Membrane Potential (mV)	
	Ventricular-Like Model Cell ^1^	Atrial-Like Model Cell ^1^
Meijer van Putten et al. [5]	−79.9	−79.2
Rocchetti et al. [15]	−78.1	−77.6
Bett et al. [14]	−79.1	−79.5
Jara-Avaca et al. [16]	−79.8	−81.0

^1^ Ventricular-like and atrial-like hiPSC-CM model cells of Paci et al. [30,31].

**Table 4:** Action potential parameters of hiPSC-CM model cell upon injection of synthetic *I*_K1_.

Action Potential Parameter	Meijer van Putten et al. *I*_K1_		Rocchetti et al. *I*_K1_		Bett et al. *I*_K1_	
	Vent	Atr	Vent	Atr	Vent	Atr
MDP (mV)	−83.9	−84.0	−79.5	−79.4	−82.2	−82.8
V_max_ (V/s)	129.1	209.6	87.0	149.8	130.4	210.0
APA (mV)	134.8	138.2	120.3	129.2	132.1	136.2
AP plateau (mV)	112.4	110.8	107.0	106.0	101.7	100.4
APD_20_ (ms)	30.8	20.8	42.4	31.5	14.8	8.7
APD_50_ (ms)	240.9	135.1	248.5	138.3	94.8	65.8
APD_90_ (ms)	306.6	183.3	311.5	189.6	144.4	96.5

Data obtained with ventricular-like and atrial-like hiPSC-CM model cells of Paci et al. [30,31]. Vent: ventricular-like cell; Atr: atrial-like cell; MDP: maximum diastolic potential; V_max_: maximum upstroke velocity; APA: action potential amplitude; AP plateau: AP plateau amplitude; APD_20_, APD_50_, and APD_90_: action potential duration at 20%, 50%, and 90% repolarisation, respectively.

## Abbreviations

4-AP	4-aminopyridine
AF	Atrial fibrillation
AP	Action potential
APA	Action potential amplitude
APD	Action potential duration
APD_20_	Action potential duration at 20% repolarisation
APD_50_	Action potential duration at 50% repolarisation
APD_90_	Action potential duration at 90% repolarisation
CTRL	Control
CV	Coefficient of variation
*E*_K_	Potassium equilibrium potential
hESC-CM	Human embryonic stem cell-derived cardiomyocyte
HEK-293	Human embryonic kidney 293
hiPSC	Human induced pluripotent stem cell
hiPSC-CM	Human induced pluripotent stem cell-derived cardiomyocyte
*I*_Ca,L_	L-type calcium current
*I*_f_	Hyperpolarisation-activated cyclic nucleotide-gated funny current
*I*_K,ACh_	Acetylcholine-activated potassium current
*I*_K1_	Inward rectifier potassium current
*I*_Kur_	Ultrarapid delayed rectifier potassium current
*I*_to1_	Transient outward potassium current
*I*_Na_	Fast sodium current
Kir2.1	Inward rectifier potassium channel subfamily 2 member 1
MDP	Maximum diastolic potential
MEA	Multi-electrode array
RA	All-trans retinoic acid
RMP	Resting membrane potential
RT-Linux	Real-Time Linux
V_m_	Membrane potential
V_max_	Maximum upstroke velocity
